# Drug-induced liver injury due to potassium iodide during thyroid storm

**DOI:** 10.1210/jcemcr/luag053

**Published:** 2026-04-21

**Authors:** Noriyoshi Takano, Yasufumi Seki, Daisuke Watanabe, Atsuhiro Ichihara

**Affiliations:** Division of Hormonal Medicine and Bioregulatory Science, Department of Medicine, Tokyo Women’s Medical University, Tokyo 162-8666, Japan; Division of Hormonal Medicine and Bioregulatory Science, Department of Medicine, Tokyo Women’s Medical University, Tokyo 162-8666, Japan; Division of Hormonal Medicine and Bioregulatory Science, Department of Medicine, Tokyo Women’s Medical University, Tokyo 162-8666, Japan; Division of Hormonal Medicine and Bioregulatory Science, Department of Medicine, Tokyo Women’s Medical University, Tokyo 162-8666, Japan

**Keywords:** allergy, drug lymphocyte stimulation test, Graves disease, thyroxine, thyrotoxicosis

## Abstract

Thyroid storm is a life-threatening condition causing multiorgan failure, including liver dysfunction. Potassium iodide (KI) is one of the key drugs used to control thyroid hormone levels. While thionamides sometimes cause drug-induced liver injury (DILI), KI has not been reported to cause DILI. Herein, we report a case of likely KI-induced DILI during treatment for thyroid storm due to Graves disease. A 30-year-old man presented with thyrotoxicosis due to Graves disease, with hyperbilirubinemia and elevated liver transaminases. The patient was diagnosed with thyroid storm, and treatment was initiated with methimazole, KI, corticosteroids, and beta-blockers. Although the thyroid hormone levels improved during the treatment, total bilirubin levels increased to 32.4 mg/dL (SI: 553 µmol/L) (reference range, <1.2 mg/dL [SI: <20.5 µmol/L]). DILI was suspected, and methimazole was discontinued; however, bilirubin levels remained elevated. Plasma exchange also failed to improve the liver injury. After KI was discontinued, the bilirubin levels rapidly decreased. Lymphocyte transformation tests for methimazole and KI were negative and positive, respectively, confirming the diagnosis of KI-induced DILI. This case highlights that KI should be recognized as a potential cause of DILI in patients with KI therapy who present with abnormal liver function.

## Introduction

Thyroid storm is a life-threatening condition caused by thyrotoxicosis, which affects multiple organ systems, including the cardiovascular, nervous, and gastrointestinal systems. Thyroid storm requires immediate interventions, including antiadrenergic drugs, potassium iodide (KI), thionamides, and glucocorticoids [1]. KI is used as adjuvant therapy for thyroid storm due to its rapid inhibitory effect on thyroid hormone release through the Wolff–Chaikoff effect [1]. End-organ damage caused by thyroid storm is usually resolved by achieving normalization of thyroid hormone levels.

Liver failure is a rare manifestation in thyroid storm. The prevalence is unknown, but some fatal cases have been reported [2, 3]. Thionamides have been recognized as possible causes of liver injury during thyroid hormone excess [4]. Iodine-containing drugs, such as iodine-131 [3] and amiodarone [5], have also been reported as a cause of liver injury. However, KI has not been reported to cause liver dysfunction.

Herein, we report a case of thyroid storm and liver dysfunction, which was drug-induced liver injury (DILI) likely caused by KI.

## Case presentation

A 30-year-old Japanese man with no prior history of hepatic or thyroid disease and with no regular medications presented with a 2-day history of fatigue, vomiting, abdominal pain, diarrhea, and palpitations (day 1). At the referring hospital, the patient presented with elevated thyroid hormone levels, with a free triiodothyronine (T3) level of 19.73 pg/mL (SI: 30.31 pmol/L) (reference range, 2.4-4.0 pg/mL [SI: 3.7-6.1 pmol/L]), a free thyroxine (T4) level of >6.00 ng/dL (SI: >77.23 pmol/L) (reference range, 0.94-1.60 ng/dL [SI: 12.10-20.60 pmol/L]), and a thyroid-stimulating hormone (TSH) level of <0.005 mIU/L (reference range, 0.610-4.230 mIU/L), indicating severe thyrotoxicosis. Liver function tests showed elevated liver enzymes and hyperbilirubinemia, with an aspartate aminotransferase (AST) level of 88 U/L (reference range, 13-33 U/L), an alanine transaminase (ALT) level of 145 U/L (reference range, 6-30 U/L), and a total bilirubin (T-Bil) level of 10.6 mg/dL (SI: 181.3 µmol/L) (reference range, <1.2 mg/dL [SI: < 20.5 µmol/L]). White blood cell count was 5.5 × 10^3^/μL (reference range, 3.3-8.6 × 10^3^/μL). The patient was diagnosed with Graves disease due to thyrotoxicosis and increased TSH receptor antibody of 12.6 IU/L (reference range, <2.0 IU/L). The patient was diagnosed with thyroid storm at the referring hospital, based on the Burch–Wartofsky Point Scale with a total score of 60 points, including tachycardia of 111 bpm (10 points) and significant gastrointestinal dysfunction with nausea, vomiting, diarrhea, abdominal pain, and jaundice (50 points). Treatment was initiated with oral methimazole 45 mg/day, oral KI 150 mg/day, oral bisoprolol 2.5 mg/day, and intravenous hydrocortisone 300 mg/day. Two days after the presentation (day 3), the patient was introduced to our hospital for further specialized endocrine and hepatology care.

**Figure 1:**
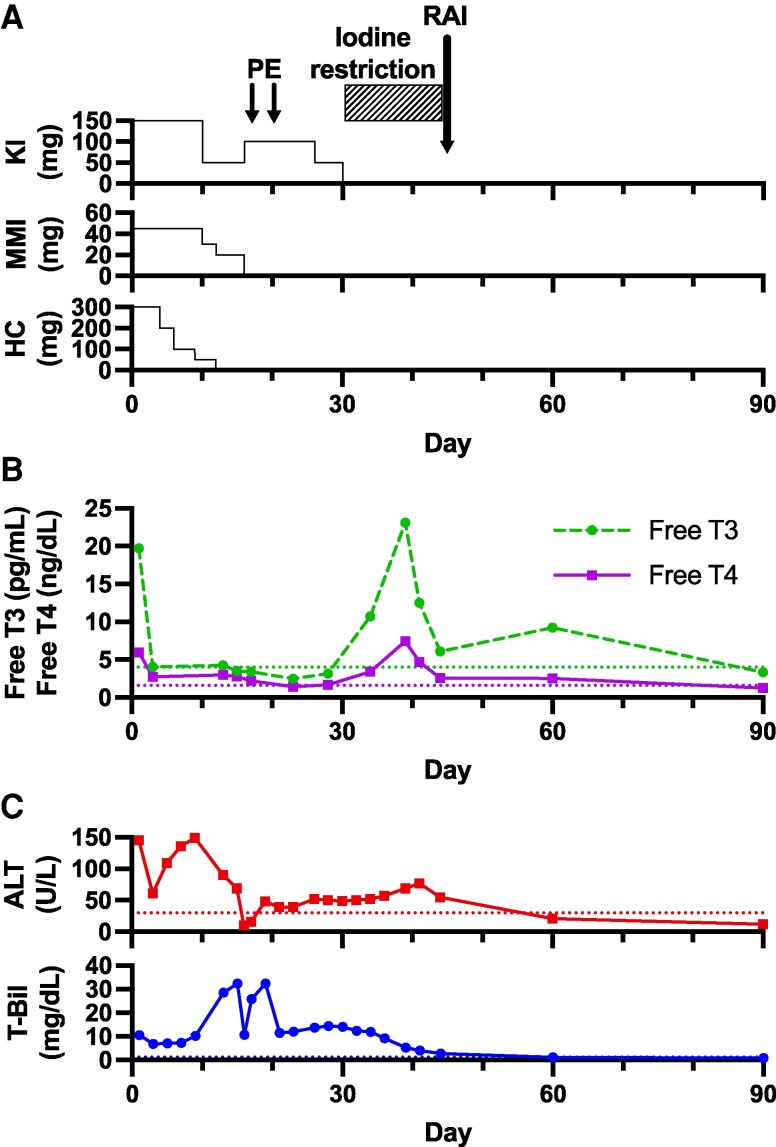
Clinical course and laboratory parameters during thyroid storm treatment. A, Treatment timeline showing doses of potassium iodide (upper panel), methimazole (middle panel), and hydrocortisone (lower panel). Above the panels, the 2 sessions of plasma exchange (days 18 and 20) and RAI therapy (day 45) are indicated. B, Serum free triiodothyronine (T3) (circles and dashed lines) and free thyroxine (T4) (rectangles and solid lines) levels. The dotted lines represent upper limits of free T3 and free T4 levels. C, Serum alanine transaminase (ALT) (upper panel) and T-Bil (lower panel) levels. The dotted lines represent their upper limits. Normal reference ranges are as follows: free T3, 2.4-4.0 pg/mL (SI: 3.7-6.1 pmol/L); free T4, 0.94-1.60 ng/dL (SI: 12.10-20.60 pmol/L); ALT, 6-30 U/L; T-Bil, < 1.2 mg/dL (SI: < 20.5 µmol/L). Abbreviations: ALT, alanine transaminase; HC, hydrocortisone; KI, potassium iodide; MMI, methimazole; PE, plasma exchange; RAI, radioactive iodine; T-Bil, total bilirubin; T3, triiodothyronine; T4, thyroxine.

On presentation, vital signs were as follows: body temperature, 36.8 °C; blood pressure, 128/76 mmHg; pulse rate, 88 bpm; respiratory rate, 16 breaths/min. Physical examination revealed conjunctival icterus, but no palpable goiter or exophthalmos was observed. Abdominal examination revealed no hepatomegaly, splenomegaly, or ascites. There was no pretibial edema. Cardiovascular and respiratory examinations were unremarkable. Laboratory findings included thyrotoxicosis, hyperbilirubinemia, and elevated liver transaminases as follows: serum free T3, 4.03 pg/mL (SI: 6.19 pmol/L); free T4, 2.75 ng/dL (SI: 35.40 pmol/L); TSH, <0.005 mIU/L; T-Bil, 6.8 mg/dL (SI: 116.2 µmol/L); AST, 36 U/L; and ALT, 61 U/L. The clinical course of thyroid function and liver function parameters during treatment is shown in Fig. 1.

## Diagnostic assessment

A gastroenterology consultation was obtained, and we investigated the cause of liver dysfunction. Liver transaminases and bilirubin levels were elevated, but the markers for viral hepatitis and autoimmune liver diseases (anti-nuclear antibodies, anti-mitochondrial antibodies, and anti-smooth muscle antibodies) were negative. Imaging studies, including abdominal sonography and computed tomography, were also unremarkable. Considering these results, the hyperbilirubinemia and elevated liver transaminases suggested acute liver injury associated with thyroid storm at the time.

The liver transaminases and bilirubin levels remained elevated despite the improvement of thyroid hormone levels following the treatment with methimazole, KI, and hydrocortisone (Fig. 1). DILI was suspected due to persistent hyperbilirubinemia despite improvement in thyroid status [2, 6]. As methimazole is a well-recognized cause of DILI [4] and was initially suspected to be a potential cause of DILI, the methimazole therapy was discontinued on day 15. At the time of methimazole discontinuation, thyroid function remained well-controlled with a free T3 level of 4.25 pg/mL (SI: 6.53 pmol/L) and a free T4 level of 2.97 ng/dL (SI: 38.23 pmol/L). Paradoxically, liver function tests had worsened despite the controlled thyroid status, with an ALT level of 90 U/L, an AST level of 48 U/L, and a markedly elevated T-Bil level of 23.2 mg/dL (SI: 396.6 µmol/L). The T-Bil level further increased to 32.4 mg/dL (SI: 553.7 µmol/L) on day 19. The lymphocyte transformation test (LTT) for methimazole was negative (stimulation index, 96% [threshold, >181%]).

## Treatment

Ursodeoxycholic acid (300 mg/day) was started for liver dysfunction. Plasma exchange was also performed on days 20 and 22, but T-Bil levels remained elevated, with the values of 10 to 25 mg/dL.

Radioactive iodine (RAI) therapy was planned for Graves disease rather than thyroidectomy because the patient preferred nonsurgical management after informed discussion of treatment options. KI was discontinued on day 30 for iodine restriction needed before RAI therapy, resulting in a rapid decline of bilirubin levels (Fig. 1).

## Outcome and follow-up

RAI therapy on day 45 successfully normalized free T3 (3.33 pg/mL [SI: 5.12 pmol/L]), free T4 (1.27 ng/dL [SI: 16.35 pmol/L]), and T-Bil (0.9 mg/dL [SI: 15.4 µmol/L]) levels by day 90. LTT for KI was positive (stimulation index, 288% [threshold, >181%]), confirming the diagnosis of DILI caused by KI. The RECAM-J 2023 score, a scoring system for diagnosing DILI, was 7 points, supporting the diagnosis of KI-induced DILI [7].

## Discussion

We report a case of thyroid storm in which DILI likely caused by KI resulted in severe hyperbilirubinemia. The patient presented with thyrotoxicosis due to Graves disease, as well as hyperbilirubinemia and elevated liver transaminases, consistent with thyroid storm. Liver dysfunction remained even after improvement of thyroid hormone levels and cessation of methimazole. Discontinuation of KI treatment before RAI therapy resulted in a rapid attenuation of bilirubin levels. A positive result of LTT for KI confirmed the diagnosis of DILI caused by KI. To the best of our knowledge, this is the first reported case of DILI potentially caused by KI with unique pathophysiological features.

The temporal relationship between drug discontinuation and improvement in liver function tests provided important diagnostic clues in our case. In patients with Graves disease, liver injury can be caused not only by thionamides but also by thyrotoxicosis itself [7]. In methimazole-induced DILI, liver injury recovers rapidly once methimazole is stopped [8], usually within a week [9]. In thyrotoxic liver injury, AST and ALT levels normalize in more than 80% of cases following euthyroidism restoration [7]. In our patient, liver dysfunction was present before the administration of methimazole and KI, but persisted from days 15 to 30 despite improvement of thyrotoxicosis and discontinuation of methimazole. After KI discontinuation on day 30, hyperbilirubinemia improved. This temporal pattern suggested a transition from initial thyrotoxicosis-induced liver dysfunction to predominant KI-induced DILI.

DILI is typically classified as direct, idiosyncratic, and indirect injury [2]. Direct DILI is caused by agents that are intrinsically toxic to the liver and is common, predictable, and dose-dependent. Idiosyncratic DILI is caused by agents that have little or no intrinsic toxicity and is unpredictable and not dose-dependent. Indirect DILI is caused by the action of the drug rather than by its toxic or idiosyncratic properties and can represent induction of a new liver condition or an exacerbation of a preexisting condition [2]. In our case, DILI was unpredictable as KI has not been reported to cause DILI. The dose of KI was lower than that recommended by the American Thyroid Association Guidelines [1], indicating the mechanism of DILI in our case may not be dose-dependent. A review summarized that LTT, which was positive in our case, had a sensitivity of 56%, a specificity of 94%, a positive predictive value of 92%, and a negative predictive value of 63% [10]. A recent study for DILI also showed that LTT had high accuracy, with a sensitivity of 77% and a specificity of 100% [11]. These findings suggest that DILI in our case can be classified as idiosyncratic DILI.

Our case suggests that KI can cause DILI in patients with thyroid storm. Liver dysfunction is a common complication with Graves disease. A systematic review reported that the prevalence of abnormal liver blood tests and elevated T-Bil level in untreated Graves disease was 60% and 12%, respectively [7]. Accordingly, when liver dysfunction persists after initiating treatment with thionamides and KI for thyrotoxicosis, it is difficult to determine whether it is due to thyrotoxicosis itself or represents DILI from the treatment. While both propylthiouracil and methimazole are recognized as possible causes of liver injury during treatment for thyrotoxicosis [4, 12], iodine-induced DILI has not been reported. As RAI therapy using iodine-131 did not worsen the DILI in our case, KI was suggested to be the cause of DILI rather than iodine itself.

Our case also suggested that the mechanism of KI-induced DILI may be associated with immune-related reactions. The mechanisms of idiosyncratic DILI have been hypothesized to involve immune hypersensitivity reactions through activation of CD4+ T cells or cytotoxic CD8+ T cells [13]. LTT, which was positive in our case, is also based on the activation and expansion of drug-specific memory T cells after an in vitro incubation of the patient's peripheral mononuclear cells with different concentrations of the suspected drugs [11]. Therefore, KI may have activated T cells, leading to immune hypersensitivity and idiosyncratic DILI in our case.

This case illustrates a critical therapeutic paradox in thyroid storm management: KI, a cornerstone therapy for controlling thyroid hormone excess in life-threatening hyperthyroidism, may paradoxically cause DILI if our report can be confirmed in clinical studies. While KI is considered essential for rapidly decreasing thyroid hormone levels [1], the concurrent development of severe hepatotoxicity created a challenging clinical dilemma. Careful monitoring of clinical course and serial assessment of liver function and thyroid hormones is crucial for the early detection of KI-induced DILI in patients with thyroid storm. In our case, it was difficult to determine whether the liver injury was attributable to iodine itself or to pharmaceutical excipients within the drug formulation. Further case reports and systematic studies are needed to clarify the causal relationship between KI administration and the development of DILI.

In conclusion, we presented a rare case of DILI potentially caused by KI during thyroid storm. As KI-induced DILI may mimic thyroid storm-induced liver dysfunction, clinicians should consider the possibility of KI-induced DILI in patients with liver dysfunction during thyroid storm.

## Learning points

- Iodine-containing drugs can cause DILI and present elevated liver enzymes and hyperbilirubinemia.
- KI may be a cause of DILI.
- KI-induced DILI should be considered as a potential cause of liver dysfunction in patients with thyrotoxicosis.
- Plasma exchange for thyroid storm may have a limited efficacy for KI-induced DILI.
- A multidisciplinary approach involving endocrinologists, hepatologists, and critical care specialists may be required to manage patients with thyroid storm.

## Data Availability

Original data generated and analyzed during this study are included in this published article.
